# Patient and health worker experiences of differentiated models of care for stable HIV patients in Malawi: A qualitative study

**DOI:** 10.1371/journal.pone.0196498

**Published:** 2018-07-19

**Authors:** Margaret L. Prust, Clement K. Banda, Katie Callahan, Rose Nyirenda, Frank Chimbwandira, Thokozani Kalua, Michael Eliya, Peter Ehrenkranz, Marta Prescott, Elizabeth McCarthy, Elya Tagar, Andrews Gunda

**Affiliations:** 1 Applied Analytics Team, Clinton Health Access Initiative, Inc., Boston, MA, United States of America; 2 Clinton Health Access Initiative, Inc., Lilongwe, Malawi; 3 HIV, TB and Health Financing Team, Clinton Health Access Initiative, Inc., New York, NY, United States of America; 4 Department of HIV and AIDS, Ministry of Health, Lilongwe, Malawi; 5 Bill & Melinda Gates Foundation, Seattle, WA, United States of America; International AIDS Society, SWITZERLAND

## Abstract

**Introduction:**

Several models of differentiated care for stable HIV patients on antiretroviral therapy (ART) in Malawi have been introduced to ensure that care is efficient and patient-centered. Three models have been prioritized by the government for a deeper and broader understanding: adjusted appointment spacing through multi-month scripting (MMS); fast-track drug refills (FTRs) on alternating visits; and community ART groups (CAGs) where rotating group members collect medications at the facility for all members. This qualitative study aimed to understand the challenges and successes of implementing these models of care and of the process of patient differentiation.

**Methods:**

A qualitative study was conducted as a part of a broader process evaluation in 30 purposefully selected ART facilities between February and May 2016. Semi-structured, in-depth interviews with 32 health workers that managed and coordinated ART clinics and 30 focus groups were held with 216 ART patients. Interviews and focus groups were audio recorded, transcribed, and coded thematically.

**Results:**

Participants reported that the models of differentiated care have yielded key benefits, including: reduced patients’ travel and visit time, decongestion of facilities, and enhanced social support. Participants suggested that these benefits could lead to improved HIV treatment outcomes for patients. At the same time, some challenges were reported, such as inconsistent stocks of drugs, which can inhibit implementation of MMS. For CAGs, the group-based nature of the model presented some unique problems, such as conflicts within groups or concerns about privacy. Health workers also described some of the reasons why eligible patients may not receive the models or conversely why ineligible patients sometimes get the models.

**Conclusions:**

Documenting patient and health worker perspectives on models of differentiated care is critical to understanding and improving these models. While these models can offer important benefits, the models may not be appropriate for all sites or patients, and patient status and needs may change over time. Key challenges should be recognized and addressed for optimal utilization of the models.

## Introduction

Not all HIV treatment patients have the same types of needs, and the movement toward differentiated models of HIV care acknowledges that, by tailoring services to different patient types, it may be possible to improve service quality and efficiency from both the patient and health system perspective [1,2]. Previous guidelines for antiretroviral therapy (ART) were developed at a time when a large portion of the ART patient population had severe clinical manifestations of disease and many ART drugs had serious side effects. However, several factors have lead to an increase in the proportion of clients on HIV treatment that are stable and healthy. Specifically, eligibility to start HIV treatment has expanded significantly in recent years [3], ART programs in many countries have been operating for many years and are very established, and ART drugs have improved, leading to fewer side effects. In this context, models of differentiated care are an important tool to ensure the provision of patient-centered care [4,5,6].

In Malawi, several models have been developed to provide more streamlined services to patients that are stable. The MOH and other in-country stakeholders sought to understand more about three models of differentiated care that were already widespread or had the potential for national scale-up. These included two facility-based models focused on individual clients, multi-month scripting (MMS) and fast-track refills (FTR), as well as one group-based, community-level model, community ART groups (CAGs). In the MMS model, stable patients are given three-month refills rather than one-month refills. For FTRs, stable patients are also given three-month refills, but only two of their four visits are required to be clinical visits with a nurse or doctor. The other two visits in the year are refill-only visits where medications are dispensed by a lower-level health worker. CAGs, which have also been launched in other countries [7], are peer-led groups that meet monthly at the community level for ARV distribution and peer-led discussions. Each month a different group member visits the facility to pick up ARV refills for the entire group, with each person having at least two opportunities for a twice-annual clinical visit.

Currently in Malawi, patients are eligible for MMS if they: are 18 years or older, have been on ART at least six months, are on first-line ART, have no adverse drug reactions or opportunistic infections, have a viral load less than 1000 copies per mL and have good adherence. Patients are eligible for FTRs and CAGs if they meet those same criteria and are also not pregnant or lactating.

Systematic assessments of the implementation of these models in Malawi were lacking, and more evidence was needed about the models to inform decision-making around model scale-up and improvement. Through a process evaluation, we sought to assist policy makers in defining the existing models, understanding the extent to which patients in Malawi were differentiated according to clinical stability criteria, and analyzing the characteristics and costs of the models of differentiated care they received. The purpose of this paper is to describe the qualitative component of the process evaluation that explored patients and provider perspectives on the key benefits and challenges associated with models of differentiated care for stable patients.

In other settings, researchers have explored the acceptability of differentiated models of care from the patient and health worker perspective. CAGs in Mozambique, similar to those implemented in Malawi, were suggested by patients in 2012 to offer cost and time savings, improved ART access, and enhanced peer support [8], and in 2015, CAG participants in Malawi reported similar benefits, along with implementation challenges related to low awareness about CAGs, patient disclosure concerns, and conflicts among CAG members [9]. Another model for integrated HIV and non-communicable disease adherence clubs has been found to be generally acceptable to patients in Kenya, but challenges with recruitment, patient understanding of the model, and practical implementation of the program emerged through interviews and focus groups [10]. Understanding and documenting these challenges, along with patient and provide input on potential benefits and general acceptability, is important for expanding and improving these models of care. There has been limited qualitative research focused on the FTR model and the somewhat more simple, but widespread, MMS model, and patient and provider perspectives on this model are critical to understand.

## Methods

This qualitative study was part of a broader mixed methods process evaluation conducted in 30 purposefully-selected facilities [11] to assess the government-led implementation of these models of care. These models were implemented by the government and partners, and the purpose of this observational was to describe and understand the current models better. For the qualitative aspect of the study, data was collected between February and May 2016 by two teams of five data collectors trained in qualitative methods.

### Ethical approval

This study received ethical approval from the Malawi National Health Sciences Research Committee (approval number 1513) and from the Chesapeake IRB in the United States (approval number 00016180).

### Sampling

We used heterogeneity sampling to achieve diversity of facilities in the sample based on: model of differentiated care offered, region, patient volume, facility owner (i.e. MOH or Christian Health Association of Malawi [CHAM]), facility type, and implementing partner support. Within each facility, one interview was conducted with the health worker in charge of the ART clinic. Health workers were therefore sampled based on those who held the specific title “ART in-charge”. In two sites, an implementing partner-supported clinical mentor was also interviewed separately. One patient focus group was conducted in each of the 30 study sites. Among the 30 sites, all were implementing MMS, 8 were implementing CAGs along with MMS, and 4 were implementing FTRs with MMS.

The facility staff members were asked to support recruitment of four to eight patients from among those that were visiting for a regularly-scheduled ART appointment. Health workers were asked to refer all willing and eligible patients to the study team at the end of their appointment, until we reached the point when we had a sufficient number of participants. Patients were eligible for inclusion in the focus group if they received one of the relevant ART models of differentiated care offered at their health facility (though since some facilities were offering multiple models, participants were included that were accessing one model but not all models offered by that site). In conversations with health workers, the study team emphasized that the questions were about the models of care and did not constitute a staff or facility performance review, so as to reduce potential for health workers to bias the sample by choosing only patients that would provide positive input.

### Data collection

Interviews and focus groups asked questions about the model process, health worker and patient satisfaction with the models, and any challenges faced. Interviews were conducted in either English or local languages. Focus groups were conducted in local languages (Chichewa, Tumbuka and Yao). Written informed consent was obtained for all participants, and participants were not compensated.

All interviews were documented through field notes and audio recordings. Achievement of theoretical saturation [12] was assessed through daily debriefings of individual data collection teams and several collective debriefings with the whole study team to discuss findings and areas lacking in clarity. Theoretical saturation for each of the three models of care was specifically assessed in terms of whether any new concepts were continuing to appear or required further development, and responses unique to each model of care were assessed individually. Additionally, transcription of audio files and initial analyses were carried out in parallel with the data collection, allowing study investigators to provide feedback to data collectors on areas requiring further exploration to achieve theoretical saturation. The study team made plans to potentially expand data collection if theoretical saturation was not reached by end of planned data collection in the 30 sites, but this was determined not to be necessary based on study procedures described here to assess theoretical saturation.

### Data analysis

Audio recordings from the interviews and focus groups were transcribed. Sessions that took place in a language other than English were simultaneously translated into English. Three members of the research team systematically coded the transcripts, using the constant comparative method [13,14]. Initially, codes were established based on themes explored in the interview guides (such as satisfaction or challenges with the models or drug storage) and then during the coding process additional codes or sub-codes were added based on the key concepts emerging from participant responses. The same coding structure was used for both health worker interviews and patient focus groups but some themes were only explored in interviews (such as patient data systems and implementing partner support). The coders independently coded batches of transcripts and then met to agree upon standard themes and codes and ensure inter-coder reliability. In this way, half of the transcripts were double- or triple-coded in batches and the coding key was adjusted iteratively. When coding was consistent and no emergent themes were identified in new transcripts, the code structure was finalized. The remaining transcripts were coded by one coder and the final code structure was applied to all transcripts coded early in the process.

Dedoose software was used to facilitate coding, organization, and retrieval of information. The research team interpreted the findings according to thematic area and wrote code summary reports to document key findings. In an effort to synthesize a large volume of qualitative data and support policy makers in prioritizing the most critical information, we developed a system for weighting the challenges and benefits of the models. In many cases, the presentation of results from qualitative data in a quantitative format is not appropriate given that sampling in qualitative studies often is not designed to be broadly representative; however in large datasets or for qualitative data sampled from a broader study sample, it can be appropriate to consider code frequencies as a valuable measure [15]. In this study, the qualitative data has been organized and prioritized based on the number of participants raising the issue and the degree to which the item had the potential to influence the overall success of the model and the goals of the ART program. Specifically, each attribute was assigned a “frequency weight”, which was a measure of how often a particular issue was raised or how many participants shared the view in interviews with health care workers or focus groups with patients. A frequency weight of three reflected that the issue was raised in more than half of interviews or focus groups in sites offering the model. A frequency weight of two or one reflected that the issue was raised in 25–50% or less than 25% of sessions in sites offering the model, respectively. Additionally, each attribute was assigned an “impact weight”, which was a measure applied by the study team in consultation with the MOH (as opposed to study participants) of the degree to which the MOH believed that this item had potential to influence the overall success of the models and its goals. An overall weight was calculated as the sum of the patient and health worker frequency weights multiplied by the impact weights. These weights were used to help policymakers interpret the information from this study and prioritize areas for action.

## Results

The characteristics of participants in the final sample for each data collection method are shown in Table 1. Overall, we held 32 ART in-charges interviews and 30 focus group discussions with 216 patients. 43.1% of our focus group sample was male, compared to 36.0% of all ART patients ever enrolled in care in Malawi as of March 2016 according to government records [16]. The characteristics of the facilities in the sample are reported elsewhere [11].

**Table 1:** Characteristics of study participants, by data collection method.

Data collection tool / characteristic	N^1^	%
**ART in-charge interview participants**	**32**	
*Cadre*		
Clinical Officer	11	34.4
Medical Assistant	9	28.1
Nurse Midwife Technician	6	18.8
Nurse	6	18.8
*Years in role (mean*, *SD)*	5.6 (5.5)	
**Patient focus group participants**	**216**	
*Gender*		
Male	93	43.1
Female	123	56.9
*Age*^2^		
18 to 30	21	9.8
31 to 45	102	47.7
46 to 64	86	40.2
65 and over	5	2.3
*Years in ART care (mean*, *SD)*	5.8 (3.9)	
*Participants from sites offering…*		
Multi-month prescriptions (30 sites)	216	100.0
Fast-track refills (4 sites)	26	12.0
Community ART groups (8 sites)	62	28.7

^1^ Numbers may not sum to total due to missing data.

^2^ The age group for each participant was estimated by the note taker.

### Successes and challenges of observed models of differentiated care

The feedback received on the MMS, FTR and CAG models was generally positive and participants could confidently testify how beneficial models had been, but also had suggestions about aspects of implementation that needed further improvements.

#### Multi-month scripting

The most commonly discussed advantages of the MMS model were related to reduced burden on patients and health workers. From the health worker perspective, MMS helped to lessen workload and decongest the facilities, while still providing an appropriate level of care to patients. For patients, MMS reduced travel costs and time spent at appointments and alleviated issues with absenteeism from work for clinic appointments:

“They started giving me three bottles, it makes me happy because I think about the distance I was travelling. From where I stay to here we pay 1000 kwacha [approximately US$1.60] on public transport… Now they are giving me three bottles so I find time to rest.”–Patient, Northern Region

Many participants also suggested that by reducing the burden on patients, MMS can help improve adherence and retention. Patients described experiences of knowing that they had an appointment but not being able to go to the clinic because of challenges with missing work, impassable roads in rainy season, not having money for transport or other logistical problems. When refills were longer, patients explained that they could more feasibly maintain the designated appointment schedule. Some health workers thought that MMS encouraged good adherence because patients were aware that if they come to appointments regularly and took medication as advised, then they would be able to get a three-month refill:

“[MMS] promotes good adherence because they know that if they have a poor adherence, it means they will be taking like one month supply. It means they will be coming to clinic frequently so most of the time the multi-month refills help a lot on good adherence.”–Health worker, Central Region

At the same time, some health workers suggested that MMS could cause patients to be more likely to miss appointments because of the long length of time between appointments.

“There is an increase in the number of defaulters… because the patients are given a lot of drugs and they end up forgetting their appointment date.”–Health worker, Southern Region

Patients reported that in addition to promoting good adherence, MMS can also encourage patients not in care to seek services. As explained by the following patient, her husband is unlikely to get tested and start ART if he thinks that HIV treatment will involve monthly appointments:

“It should be 3 months because my husband really wants to come for HIV test, but he fail[s] because he is afraid of his boss. So [if] the time to refill drugs gets extended, he can come for a test and start on ART. It can really help.”–Patient, Central Region

Most patients in the focus groups explained that they felt comfortable and knew when to access more care if they needed it. Specifically, patients reported that if they felt sick between ART appointments, they would come to the clinic even if they still had ARVs remaining to get care for their symptoms. For example:

“When you get your three months medication and you have a problem even before your appointment date you have to come to the hospital that is what we are told, that we can be given many months but if you get sick you have to come to the hospital so that you get helped.”–Patient, Central Region

Although many patients reported that they would return to the clinic if they got sick between ART appointments, some health workers expressed a concern that patients were not coming back to the clinic promptly to report any problems, but rather were waiting until their next appointment date:

“We give health talks on the importance of attending OPD [out-patient department] but most of them they prefer to stay at home while sick waiting for the next appointment… They say they don’t want to visit the hospital regularly so they choose to remain at home until they finish the medication.”–Health worker, Central Region

Other challenges with implementing the MMS model were also described by patients and health workers. The most common challenge described was related to the perceived availability of ARVs and cotrimoxazole. While it is not clear whether the MMS model may have influenced stock security, participants frequently pointed out that limited drug stocks prevented full implementation of the MMS model. A patient in the Southern Region reported, “*sometimes they give us for 2 months… they tell us there is low stock of drugs*.” Based on feedback from health workers, it seems that the most commonly prescribed regimen is nearly always in stock; but many facilities experienced low stocks of other first-line regimens and second-line regimens. Additionally, patients are supposed to receive cotrimoxazole prophylaxis along with their ARVs, but stockouts and low stock levels of cotrimoxazole frequently meant that patients either did not receive cotrimoxazole or received only a small amount.

“Like the [cotrimoxazole], that affects the amount of drugs we dispense to the client sometimes if we don’t have in our stock, they go with nothing in terms of the cotrimoxazole sometimes when we have few, now we have to reduce the months, the number of months they are going to get, say a person has come for a three-months visit, they get ARVs for 3 months and cotrimoxazole for 1 month.”–Health worker, Northern Region

While some patients report returning to the facility for more cotrimoxazole even if they have ARVs, other patients simply go without the cotrimoxazole. A few patients described using traditional medicines when cotrimoxazole was not provided.

Another challenge is that the policies about MMS may be implemented differently across facilities, creating frustrations for patients that transfer between facilities. There were several accounts of patients who switched from one ART clinic to another and were confused or frustrated by differences in refill length across facilities. Several patients described receiving three-month refills at one clinic, but then transferring to a new clinic where the longest refills offered were for two months.

Some stakeholders in Malawi had concerns about whether giving larger quantities of medications at the same time would increase the possibility of medications being misused by patients, so participants were prompted to talk about how they stored their medications and whether they had ever heard of cases of ART drugs being sold or used for any purpose other than as prescribed. Patients described taking precautions to safeguard their medications by storing them in a safe place. Patients were particularly careful to make sure that children did not have access to tamper with the medications. Most participants had never heard of any form of misuse of ART drugs in their community and even responded that they were unable to imagine how or why this would happen.

“This medicine being sold? I have never heard. Maybe the other medicine [other than ARVs] and the one who sells is not sick… like me here I should take my medicine and sell? That means I want death.”–Patient, Northern Region

While there were no reported cases of patients selling ARVs, two participants reported that health workers may be involved in selling drugs outside of health facilities. Patients suggested that illegal sales of drugs must be supported by health workers because the drugs were available in full cartons that individual patients would not have access to. A few participants reported having heard rumors that ARVs were being used in brewing traditional beer to make it strong and given to domestic animals to make them grow quickly and be strong. These rumors could not be verified and were only reported by a couple of the participants.

#### Fast-track refills

Because FTRs are implemented alongside or in addition to MMS, the benefits and challenges of MMS apply also to the FTR model. In addition to the topics described in relation to MMS, there were several other points that participants brought up that are unique to the FTR model. For example, some participants from FTR sites felt that the FTR model further reduced the workload for clinical staff such as clinicians and nurses. The model allowed those clinical staff to spend more time with patients that have a complaint or problem and need more care:

“Now the system is working very well and has reduced congestion to the clinician because previously we were meeting almost 60 or 70 or even 80 something [established] patients… but now with [FTR] the clinician does meet maybe 40 patients or 35 plus new patients.”–Health worker, Southern Region

At the same time, the FTR can reduce waiting time for patients. Whereas patients previously had to wait for long period to see the clinical health workers, they now received services at some appointments from other cadres of health workers with reduced waiting time. But even with reduced waiting times, patients like this one report waiting up to 90 minutes for a fast-track refill and longer for regular appointments:

“When we come and [are] not meeting the doctor, it is faster than when we are meeting the doctor. Because you can get here at half past six, that means by the time they are opening at eight o’clock you will be the first to collect drugs. Within ten minutes you are done. But the day we are meeting nurses we take very long.”–Patient, Southern Region

While some patients described the wait times as being shorter when they did not have to see the clinician or nurse, other patients talked about how the wait time to see a Health Surveillance Assistant (HSA) was still long. In particular there were complaints about the HSA arriving later than the specified opening time, though it is possible that the same could occur if services were provided by another health worker.

“The challenge is the fact that you come as early as 5 o’clock… but they don’t open on good time to start dispensing drugs. Sometimes you find that… the one who dispenses drugs hasn’t yet arrived so you come early and you still leave late.”–Patient, Southern Region

#### Community ART groups

CAGs transfer some aspects of care from the facility to the community level, so that patients do not have to visit the ART clinic as frequently. For patients in rural areas that may live far away from the clinic, this was a major benefit. In addition to serving as an ARV delivery mechanism, the CAGs serve an important role in terms of increasing social support among people living with HIV. One patient from the Southern Region described the relationships within the CAG as follows: “*We are like one family*, *we advise each other and encourage each other*.” In addition to providing social support, the CAGs can form the foundation for other types of material support among members. This is not a formal part of the CAG program and is not promoted by facility staff, but can occur as the initiative of the members themselves:

“We meet twice a month, we contribute money for emergencies if one of us is sick we go visit. And help with food or transport to the hospital … It is going well and we rest now and work normally without coming here often.”–Patient, Southern Region

While many patients reported positive perceptions of and experiences with CAGs, the model was not a good fit for every patient. For example, participants in the focus groups and interviews talked about some challenges with CAGs, such as concerns about joining a CAG due to privacy and confidentiality issues.

“I am not in a group. I feel there is no privacy. When you go to the groups everyone knows you are on medication which makes it very difficult for us with the discrimination.”–Patient, Southern Region

The fact that some patients had concerns about joining a CAG made it difficult for others to form a group in some cases. Some individuals were eager to be a part of a CAG but they did not know others in their community with HIV or were not willing to approach others about forming a group:

“I would [join a CAG] but we don’t have a lot of people who are willing to start in our village. People who are interested are not from our village, three from another village and two from the other… I think most people hide themselves.”–Patient, Southern Region

Even for patients that were willing to join a CAG, they sometimes faced problems once the group was established. As with any group-based process, there were sometimes inter-personal conflicts or issues among members. One patient described how some members of the group did not follow the guidelines and created problems for other members:

“Sometimes we have problems like other people when it is their turn to collect drugs for the rest of the members in the group; they send their children to collect for them which is not allowed.”–Patient, Southern Region

Some patients also made statements that indicated that they may not have fully understood the guidance provided to them about how the CAG should work. According to the CAG protocols, if a member of the group is feeling sick, then that member should go to the clinic for the appointment regardless of whether it is their turn. If needed, that patient may be accompanied by the person whose turn it is for support. However, several patients described one of the benefits of CAGs being that they did not have to go to the clinic when they felt too sick to travel. This indicates that patients have not fully understood the guidance provided about CAGs.

“We think the groups are good. One of us could get sick and still receive the drugs without going to the hospital… Today the one who was supposed to come is not feeling well, so I volunteered to come and collect the drugs.”–Patient, Southern Region

Other patients recognized that if a group member was sick on or near the appointment date, that member was supposed to go for the appointment, and if that patient required help to get to the facility, a group member could accompany them. Still, some health workers expressed concerns about the reduced frequency of monitoring or seeing patients at the ART clinic. Despite the role that the rest of the group plays in supporting each other to recognize symptoms of treatment failure and get care, there continue to be some concerns that patients may develop problems or have reduced adherence and that these problems would fail to be recognized by health workers until they had progressed to a severe stage.

#### Prioritization of benefits and challenges

We prioritized the key advantages and challenges associated with each model as shown in **Table 2**. This information should not be used to compare across models, but rather to prioritize issues for consideration regarding each individual model.

**Table 2:** Weighting of benefits and challenges of differentiated models of care^1^.

Model of Care / Type of Feedback Provided	Frequency weighting^2^ by health workers	Frequency weighting^2^ by patients	Impact weighting^3^ according to policy makers	Overall weighting^4^
**MMS**				
**Successes and benefits**	** **	** **	** **	** **
Reduced burden on patients	2	3	4	20
Reduced workload for health workers and congestion in facilities	2	1	5	15
Improves patient adherence and retention due to reduced burden of care	3	0	4	12
Acts as incentive for good adherence or to seek services	1	1	4	8
Helps patients to maintain confidentiality	0	1	2	2
Patients felt comfortable seeking services between appointments if needed	0	1	3	3
**Challenges**	** **	** **	** **	** **
Low stocks of ARVs	1	2	5	15
Patients failing to seek care when sick between appointments	2	0	5	10
Perception that patients are more likely to forget spaced out appointments	2	0	5	10
Low stocks and stock outs of cotrimoxazole limiting implementation	1	2	3	9
Preference for refills of longer than three months	0	3	2	6
Differences in implementation across facilities	0	1	2	2
**FTR**^5^				
**Successes and benefits**	** **	** **	** **	** **
Reduced waiting time for patients	0	3	5	15
Reduced workload for clinicians	2	1	4	12
**Challenges**	** **	** **	** **	** **
Transitioning patients out of FTR program when they become unstable	2	0	4	8
Lack of patient understanding of model of care	1	0	3	3
Perceived long waiting time for refill visits	0	1	2	2
**CAG**				
**Successes and benefits**	** **	** **	** **	** **
Improves patient adherence and retention in care due to social support	3	2	4	20
Reduced travel time and burden	0	3	4	12
Encouragement of members to seek care when sick	1	1	4	8
Increased social support	0	2	4	8
Reduced space issues and facility congestion	2	0	3	6
Development of insurance and other financial support systems	0	1	1	1
Challenges	** **	** **	** **	** **
Misunderstandings or relationship problems within groups	3	2	3	15
Limited ability for health workers to monitor patient adherence and status	3	0	4	12
Patient concerns about privacy	2	2	3	12
Transitioning patients out of CAG program when they become unstable	2	0	3	6
Difficulties in establishing group	0	2	3	6
Lack of education for and understanding of patients about CAG model	0	2	3	6
Lack of training for and understanding of health workers about CAG model	1	0	4	4
Lack of resources for supervision	1	0	4	4
Perceived low male participation	1	0	1	1

Notes

1. This table is based on qualitative data from the process evaluation and consultation with government officials about the impact or importance of each item. Because of the nature of qualitative data collection as in-depth, guided conversations on a topic, it is not possible to take a strictly quantitative approach to assessing the issues raised. Rather, we have attempted to give a general weighting for how often an item was raised in qualitative data and how important the item is to the success of the model, according to the MOH.

2. Frequency Weighting: This is a measure of how often a particular issue was raised or how many participants shared the view in interviews with health care workers (HCWs) or focus groups with patients. A frequency weighting of three reflects that the issue was raised in more than half of interviews or focus groups in sites offering the model. A frequency weighting of two or one reflect that the issue was raised in 25–50% or less than 25% of sessions in sites offering the model, respectively.

3. Impact Weighting: This is a measure applied by the study team in consultation with the MOH (as opposed to study participants) of the degree to which policy makers believed that item had potential to influence the overall success of the models and its goals if the issues were to be realized.

4. Overall Weighting: The overall weighting is calculated as the sum of the patient and health worker frequency weightings multiplied by the impact weighting.

5. Note that the benefits and challenges of MMS apply to FTRs also. But under FTRs the only benefits and challenges listed are the ones that are in addition to or different from the MMS issues.

### Process of patient differentiation

Regardless of the model being offered at a particular facility, these models share the characteristic of being provided differentially to patients determined to be stable. Across all models, it was acknowledged that there are some challenges in differentiating patient groups and ensuring that only those that are eligible receive the model. We explored reasons why ineligible patients were included in the models. The most common reason suggested by health workers was that patients often make special requests, especially for MMS, if they live a long distance from the facility or are travelling. This is allowed by the guidelines; the MOH ART guidelines state that “in exceptional cases (e.g. international travel) up to 6 or even 12 months of ARVs can be dispensed” [17], though the guidance is not specific as to the extent of situations that may qualify as exceptional. In many cases, were asked in interviews to describe the eligibility criteria for these models of care, they provided incorrect or incomplete responses, indicating that health worker knowledge of these eligibility criteria may be incomplete. In other cases, health workers described the desire to reduce congestion or workload in facilities as a key factor in the decision of enrolling patient in these differentiated models of care. For example, one in-charge described how their facility uses a quota system for each ART day and may give patients longer refills if they meet that quota.

“The other issue is about how many patients you have booked for a particular day … If you think you need to see 100 patients in a day, as you reach of that number, you may think of changing this particular patient. Instead of giving 2 months, I will give him 3 months so that we relieve those that will be working on that particular day.”–Health worker, MMS only site in Central Region

Finally, ineligible patients may receive these models if a transition from being eligible to ineligible is not recognized. If patients have previously been included in a model, it was suggested that some health workers may continue to include them without assessing eligibility at each visit.

## Discussion

Models of differentiated care are being successfully implemented in Malawi and patients and health workers reported that these models have yielding key benefits. All three models investigated in this study, MMS, FTRs and CAGs, can reduce the burden on health workers and patients. For patients, travel time and visit time can be reduced by fewer or shorter visits to the ART clinic. For health workers, seeing individual patients less frequently can reduce congestion in facilities. At the same time, some challenges have been faced in attempting to implement these models. For MMS and FTRs, inconsistent stocks of ART and other supplemental drugs can restrict the quantity of medications provided at each visit thereby inhibiting implementation of the models. For CAGs, the group-based nature of the model presented some unique problems, such as conflicts within groups or patient concerns about privacy. Other published literature highlights similar challenges and benefits to the CAG model in Malawi [9] and Mozambique [8] though previous studies have not pointed specifically to challenges related to transitioning patients out of the model if they become unstable or ineligible or to the issue of limited resources for supervision and implementation. This latter issue on resources for supervision has likely arisen in the more recent past since implementing partners have attempted to transition supervision of the model to the government. For all models, some health workers mentioned concerns about limited ability to identify treatment failure or side effects when patients are seen less frequently and about whether patients would report to the facility if they experienced any adverse events before their next appointment.

These results should be viewed in light of several strengths and weaknesses. This study captured a wide range of in-depth perspectives on three differentiated models of care from patients and health workers in different types of facilities. Whereas other studies cited in this work have tended to explore only one model of differentiated care at a time, this study was able to bring perspectives on multiple models together. At the same time, the facilities and participants included in this study were selected purposefully and cannot necessarily be considered to be nationally-representative. Additionally, it is important to note that these findings may not be generalizable outside of the Malawian context. Policy makers should consider the lessons of this qualitative study together with other types of evidence regarding differentiated models of care.

These results have implications both for research and practice in relation to differentiated care. In order to continually improve the implementation of these and similar models, mechanisms should be established to identify and address challenges unique to each model. In Malawi, challenges such as low stocks of ARVs and cotrimoxazole and limited resources for monitoring of CAGs should be considered by program managers since planning and proper supervision may be effective in reducing these issues [18]. Additionally, all models were impacted by challenges with accurately differentiating patients and recognizing changes in status. As suggested by other experts, the expansion of access to viral load testing may improve differentiation of patients [19]. Improvements in data system structure may also support health workers to differentiate patients.

A key question for researchers is related to the adequacy of patient outcomes for those included in these models. Participants suggested that by reducing the burden on patients to remain in care, the models may help to improve retention in care. CAGs may play a unique role in encouraging retention as well due to the social support provided by the group nature of the model. Research suggests that patient outcomes in these models are acceptable [20], but determining the impact of these models on patient outcomes such as retention or viral suppression is challenging due to the differences in the types of patients that are enrolled or not enrolled in the models. A systematic review suggested that less frequent clinic visits may be associated with improved treatment outcomes [21] but noted that the evidence base was sparse and of low quality. Researchers have considered the retention and viral suppression of patients in models similar to Malawi’s CAGs in Mozambique [22], South Africa [23], and Uganda [24] in an observational way. While rigorous analyses using a counterfactual should be explored to understand the impact of these models, randomization of clients into models such as CAGs may not be appropriate since client choice and motivation to join these models may be a critical factor in their success [25]. While it remains a priority to refine methodologies to rigorously assess the impact of these models, it is also important to continue to explore patient perspectives on these models, since the very goal of the models is to deliver patient-centered care. Future research should also focus on understanding perspectives of patients not enrolled in the models and exploring discrepancies in the perspectives of patients and health workers about these models. For example, patients reported returning to the clinic before their appointments when they felt sick, but health workers believe that patients would delay returning to the clinic. Further qualitative and quantitative investigations should assess these accounts since both patient responsibility and health worker confidence are critical to the success of these models.
